# Profiling of Glycan Receptors for Minute Virus of Mice in Permissive Cell Lines Towards Understanding the Mechanism of Cell Recognition

**DOI:** 10.1371/journal.pone.0086909

**Published:** 2014-01-27

**Authors:** Sujata Halder, Susan Cotmore, Jamie Heimburg-Molinaro, David F. Smith, Richard D. Cummings, Xi Chen, Alana J. Trollope, Simon J. North, Stuart M. Haslam, Anne Dell, Peter Tattersall, Robert McKenna, Mavis Agbandje-McKenna

**Affiliations:** 1 Department of Biochemistry and Molecular Biology, University of Florida, Gainesville, Florida, United States of America; 2 Department of Laboratory Medicine, Yale School of Medicine, New Haven, Connecticut, United States of America; 3 Department of Biochemistry, Emory University School of Medicine, Atlanta, Georgia, United States of America; 4 Department of Chemistry, University of California Davis, Davis, California, United States of America; 5 Division of Molecular Biosciences, Imperial College London, London, United Kingdom; German Primate Center, Germany

## Abstract

The recognition of sialic acids by two strains of minute virus of mice (MVM), MVMp (prototype) and MVMi (immunosuppressive), is an essential requirement for successful infection. To understand the potential for recognition of different modifications of sialic acid by MVM, three types of capsids, virus-like particles, wild type empty (no DNA) capsids, and DNA packaged virions, were screened on a sialylated glycan microarray (SGM). Both viruses demonstrated a preference for binding to 9-O-methylated sialic acid derivatives, while MVMp showed additional binding to 9-O-acetylated and 9-O-lactoylated sialic acid derivatives, indicating recognition differences. The glycans recognized contained a type-2 Galβ1-4GlcNAc motif (Neu5Acα2-3Galβ1-4GlcNAc or 3′SIA-LN) and were biantennary complex-type N-glycans with the exception of one. To correlate the recognition of the 3′SIA-LN glycan motif as well as the biantennary structures to their natural expression in cell lines permissive for MVMp, MVMi, or both strains, the N- and O-glycans, and polar glycolipids present in three cell lines used for *in vitro* studies, A9 fibroblasts, EL4 T lymphocytes, and the SV40 transformed NB324K cells, were analyzed by MALDI-TOF/TOF mass spectrometry. The cells showed an abundance of the sialylated glycan motifs recognized by the viruses in the SGM and previous glycan microarrays supporting their role in cellular recognition by MVM. Significantly, the NB324K showed fucosylation at the non-reducing end of their biantennary glycans, suggesting that recognition of these cells is possibly mediated by the Lewis X motif as in 3′SIA-Le^X^ identified in a previous glycan microarray screen.

## Introduction

Viruses serve as robust genome delivery vehicles evolved to utilize an array of intricate strategies for successful infection of target host organisms. The key initial step is virus attachment to cell surface receptors, followed by internalization into the cytosol, targeting, and release of the viral genome to the replication compartment. Proteins, carbohydrates, and lipids constitute receptors for many enveloped and non-enveloped viruses. Some serve as ‘attachment factors’ that concentrate virus on the cell surface and stimulate it to bind to secondary receptors or co-receptors that guide virus entry into cells. Glycans (complex carbohydrates) are the major constituents of the cell surface and may be conjugated to proteins or membrane lipid head groups to form glycoproteins and glycolipids, respectively, or are present as glycosaminoglycan (GAG) chains attached within proteoglycans. The variability of the glycan structures between different species and between tissues in the same species as well as their exposed position at the cell surface makes these glycoconjugates excellent targets for virus attachment [1]. For most viruses that utilize glycan receptors, the binding is mediated by electrostatic interaction between the viral attachment protein and negatively charged sialic acid (SIA)-containing glycan determinants or sulfated portions of glycosaminoglycans. The virus recognition motif can be spike glycoproteins displayed on the lipid bilayer of enveloped viruses, or specific features, such as protrusions or depressions, conformed on the assembled capsid surface of non-enveloped viruses. The motif may be composed of a single protein or assembly of several proteins. The diverse interactions that occur between the viral pathogen and host, especially the recognition of cell surface receptors determines host specificity and tissue tropism. The pathway of entry into the cell and subsequent interactions influence the pathogenic outcome of infection.

Many members of the *Parvoviridae* family, single-stranded DNA containing non-enveloped icosahedral viruses, utilize glycans for attachment or/and cellular entry. Bovine adeno-associated virus and human parvovirus B19 use gangliosides, adeno-associated virus serotype 1 (AAV1), AAV4, AAV5, AAV6, bovine parvovirus, H-1 parvovirus, porcine parvovirus, and minute virus of mice (MVM) use SIA, AAV2 and AAV3b use heparan sulfate proteoglycan, and AAV9 utilizes terminal galactose as a receptor [2]–[20]. Sialic acid commonly occurs at the non-reducing termini of glycans linked within glycoconjugates and comprises a family of structurally diverse monosaccharides derived from neuraminic acid, a nine-carbon sugar. More than 50 natural analogues result from modifications to the SIA backbone [21]. In general, the C-5 position is either N-acetylated (N-acetylneuraminic acid; Neu5Ac), N-glycolylated (N-glycolylneuraminic acid; Neu5Gc) or hydroxylated (deaminoneuraminic acid; KDN). The hydroxyl groups on SIA can be free, esterified (acetylated, lactoylated, sulfated, phosphorylated), or etherified (methylated), leading to increased chemical diversity [22].

Two strains of MVM, the prototype strain (MVMp) and the immunosuppressive strain (MVMi), serve as models for understanding the role of parvovirus capsid-SIA receptor interaction in infection. Previous studies, in which pretreatment of fibroblasts with neuraminidase or proteinase K abolished MVM infection showed that SIA-containing glycoprotein(s) are required for cellular recognition [7], [23]. This requirement was further confirmed by glycan microarray screening [24]. Nam et al. [24] showed that both MVM viruses specifically recognize α2-3 sialylated glycans with a type-2 Galβ1-4GlcNAc motif (3′SIA-LN) and a Lewis X Galβ1-4(Fucα1-3)GlcNAc motif (3′SIA-Le^x^) with MVMi showing expanded recognition of multisialylated glycans with terminal α2-8 linkages. The two viruses share 97% sequence identity in the capsid viral protein (VP) amino acid sequence and are serologically indistinguishable. MVMp was originally isolated from a murine adenovirus stock and replicates efficiently in mouse fibroblasts such as the A9 cell line, whereas MVMi, which was recovered from an EL4 T-cell lymphoma, replicates in mouse T lymphocytes and hematopoietic precursors [25]. The viruses are reciprocally restricted for growth in each other’s cell type. Cell binding assays show that both strains bind to and enter the restricted cell, but there is a block prior to replicative form DNA replication likely due to a block in uncoating which is dictated by the VP sequence [26]–[28]. Both viruses are however oncotropic and display oncolytic activity *in vitro*, and are both able to infect the transformed human cell line NB324K (SV40 transformed human newborn kidney fibroblast cells), suggesting that the restricting environment is eliminated in tumor cells. *In vivo*, following intranasal inoculation into newborn mice, MVMi is pathogenic and replicates in endothelia, neuroblasts [29], and hematopoietic stem cells. In adult Severe Combined Immunodeficient (SCID) mice MVMi causes acute leucopenia [30], while MVMp infection is asymptomatic [31]. The 3′SIA-Le^X^ motif, a known tumor cell marker [32], [33], has been proposed as the mediator of transformed cell recognition by the MVM viruses and the interaction with the multisialylated α2-8 glycans, found in brain glycoproteins [34], the mediator of MVMi’s neurotropism.

In this study, MVMp and MVMi were screened on a sialylated glycan microarray (SGM) containing terminal SIA structures presented on type-1 (Galβ1-3GlcNAcβ1-R) and type-2 (Galβ1-4GlcNAcβ1-R) glycan precursors to determine (I) if they recognize SIAs with different modifications and (II) if there is a difference in the SIA recognition and affinity by MVM capsids with a packaged genome (virions), empty (no DNA) capsids, and virus-like particles (VLPs) containing only their major capsid protein, VP2. All the viruses bound to glycans with the 9-O-methyl SIA derivative with the MVMp viruses showing additional recognition of 9-O-acetyl and 9-O-lactoyl SIA derivatives. Both MVM strains also recognized biantennary glycans with terminal 3′SIA-LN motifs. The data verified that the surface properties of VLPs are indistinguishable from those of wild type empty capsids and virions, and validates their use in lieu of infectious virions to study MVM interactions with host cell surface glycan receptors. Glycan profiling of cells reciprocally restrictive for MVMi and MVMp (A9 fibroblasts and EL4 T lymphocytes, respectively) or permissive for both viruses (NB324K), identified glycan compositions consistent with those recognized by the MVM capsids in the SGM and previous glycan microarrays [24], [35]. These observations indicate a potential role for the glycans identified by these solid-phase binding assays in cellular infection by the MVM viruses.

## Materials and Methods

### Cell Lines

A9ouab^r^11 cells are a ouabain-resistant derivative of the HGPRT^−^ L mouse fibroblast cell line A9 that are permissive host cells for MVMp [36]. EL4 T cells are a strain of the mouse T-cell lymphoma that are permissive for MVMi [36]. NB324K is a clone of simian virus-40 transformed human newborn kidney fibroblast cells that is permissive for both MVMp and MVMi [36]. Glycomic profiling was performed with 1×10^7^ cells of each mammalian cell line, which were cultured in Dulbecco’s modified Eagle medium (DMEM) supplemented with 5% heat-inactivated fetal bovine calf serum.

### Glycan Profiling

The cells were scraped into the medium and harvested by centrifugation at 250×*g* for 5 min. Cells were washed three times with 1X phosphate-buffered saline (PBS, Invitrogen, Carlsbad, CA), pelleted, resuspended in 1 ml of PBS, transferred to a microfuge tube, and pelleted at 800×*g*. The supernatant was aspirated, and the cell pellet was stored at −80°C. Further processing of the cell lines to derivatize, extract, purify, and analyze the glycans by mass spectrometry was carried out as described by North et al. [37].

### Generation of Full (MVM-Full) and Empty MVM (MVM-Empty) Capsids

A9 ouab^r^11 cells were grown in spinner culture in DMEM containing 5% fetal bovine serum and antibiotics to a density of ∼6×10^5^ cells/ml. The cultures were infected with predetermined titers of transfection-derived parvovirus MVMp stocks and expanded until cell counts indicated a progressive rise in numbers of dead cells. The cells were harvested by centrifugation, washed in PBS without Ca^2+^or Mg^2+^, pelleted, and resuspended in 10 ml of TE8.7 (50 mM Tris-HCl pH 8.7, 0.5 mM EDTA) per liter of infected cells. Following three cycles of freeze-thaw at 37°C the samples were centrifuged repeatedly at 800×*g* to clarify the supernatant and were stored at −20°C.

For virus purification, 6-ml supernatant aliquots were further clarified by centrifugation at 14,462×*g* in a Sorvall SS34 rotor at 4°C and then floated on top of a 6-ml iodixanol (OptiPrep; Axis-Shield, Oslo, Norway) step gradient (1 ml 55% and 2 ml 45% in PBS plus 1 mM MgCl_2_ and 2.5 mM KCl, followed by 2 ml 35% and 1 ml 15% in TE8.7). Samples were centrifuged at 209,491×*g* for 18 h at 18°C in a Beckman SW41 rotor. Fractions were collected from the bottom of the gradient and full and empty capsid concentrations were assessed by hemagglutination and sodium dodecyl sulfate-polyacrylamide gel electrophoresis (SDS-PAGE). The capsid integrity was checked by negative-stain electron microscopy (EM). For the EM, 5 µl of purified virus at an estimated concentration of 2.0 mg/ml was spotted onto a 400 mesh carbon-coated copper grid (Ted Pella, Inc., Redding, CA, USA) for 1 min before blotting with filter paper (Whatman No.5). The sample was then negatively stained twice with NanoW (Nanoprobe) for 1 min, blotted dry, and viewed on a Hitachi 3000 electron microscope. MVMi (full and empty) capsids were produced in the same way as MVMp except that the transfection utilized NB324K cells. For SGM screening, all viruses were buffer-exchanged into 1XPBS and the concentrations adjusted to 0.5 mg/ml using Ultrafree 150 kDa cut-off centrifugal filter units (Millipore, Billerica, MA).

### Recombinant Virus Production and Purification

Recombinant baculovirus constructs expressing the VP2 of wt MVMi and MVMp that self-assemble into VLPs (MVMp-VLP and MVMi-VLP) were constructed as described [38]. Sf9 insect cells growing in suspension culture in Erlenmeyer flasks in Sf-900 II SFM media (Gibco/Invitrogen Corporation) at 27°C were infected with a titered baculovirus construct at a multiplicity of infection of 5.0 plaque-forming units per cell. Following incubation at 27°C for ∼70 h, the cells were spun down at 1,588×*g* for 15 min in a JA-10 rotor and then the pellet resuspended in lysis buffer (50 mM Tris-HCl pH 8.0, 150 mM NaCl, 0.2% Triton X-100, 10 mM MgCl_2_). The MVMp and MVMi VLPs were purified based on published procedures with some modifications [38]. The VLPs were released from the cells by three cycles of rapid freeze-thaw with the addition of Benzonase (Merck KGaA, Germany) after the second cycle. The cellular debris was removed by low speed centrifugation at 12,064×*g*, 15 min, and 4°C in a JA-20 rotor. The supernatant was diluted with TNET buffer (50 mM Tris-HCl pH 8.0, 100 mM NaCl, 1 mM EDTA, 0.2% Triton X-100) and pelleted through a 20% (w/v) sucrose cushion by ultracentrifugation at 207,871×*g* for 3 h at 4°C in a 70Ti rotor. The resulting pellet was resuspended overnight at 4°C in TNTM buffer (25 mM Tris-HCl pH 8.0, 100 mM NaCl, 0.2% Triton X-100, and 2 mM MgCl_2_). The resuspended sample was then subjected to a low-speed spin at 800×*g* for 2 min to remove particulate material and further purified by ultracentrifugation on a sucrose-step gradient (5–40% w/v in TNTM) at 209,491×*g* for 3 h at 4°C in a Beckman SW41Ti rotor. A visible blue fraction containing VLPs, sedimenting at ∼20–25% sucrose, was extracted with a syringe and needle, and dialyzed against TE buffer (25 mM Tris-HCl pH7.5, 1 mM EDTA). Next, CsCl was added to the dialyzed sample to a final density of 1.31 g/cm^3^ and then subjected to equilibrium centrifugation at 209,491×*g* for 24 h at 4°C in a Beckman SW41Ti rotor. Visible virus fractions were extracted with a syringe and needle, and extensively dialyzed into 1XPBS. The concentration of the viruses was estimated from optical density measurements (assuming an extinction coefficient of 1.0 for MVM VLPs) and adjusted to 0.5 mg/ml using Ultrafree 150 kDa cut-off centrifugal filter units (Millipore, Billerica, MA). The purity and integrity of the viral capsids were monitored using SDS–PAGE gel electrophoresis and negative-stain EM, respectively, as described above.

### Preparation of SGM (Sialylated Glycan Microarray) and Virus Screening

Terminally sialylated glycans with various SIA modifications were synthesized as previously described [39], [40]. Seventy-seven sialylated structures were generated incorporating 16 different terminal SIAs on 4 different underlying structures that were tagged with a fluorescent linker (Table S1). Each glycan was quantified based on its fluorescence and printed on NHS-activated glass slides in replicates of *n* = 4 to generate the SGM. The printed slides were washed and blocked with 50 mM ethanolamine in 0.1 M Tris-HCl buffer (pH 9.0) for 1 h, and prior to screening the slides were rehydrated for 5 minutes in TSM buffer (20 mM Tris-HCl, 150 mM NaCl, 0.2 mM CaCl_2_, and 0.2 mM MgCl_2_). The virus samples were diluted with Binding Buffer (TSM buffer plus 1% BSA and 0.05% Tween 20) to give a final volume of 70–100 µl (0.05 mg/ml), which was added to the slide and incubated at RT for 1 h. The slides were then washed with Wash Buffer (TSM buffer plus 0.05% Tween 20), and an anti-MVM capsid antibody (Tatt-2; polyclonal from rabbit) was added at a dilution of 1∶5,000. The slides were washed again with Wash buffer and incubated with Cy5-labeled-goat anti-rabbit IgG at 5 µg/ml. The slides were scanned with a Perkin Elmer ProScanarray microarray scanner equipped with 4 lasers and for Cy5 fluorescence, the wavelengths 649 nm (Excitation) and 670 nm (Emission) were used. The scanned images were analyzed with the ScanArray Express software to determine the average relative fluorescence units (RFU) and standard deviation (S.D) of the four replicates. In the output plot, the y-axis represents the average RFU and the x-axis represents 77 sialylated glycans and seven controls (LNnT, NA2, Man5, LNT, lactose, fetuin, and biotin, see abbreviations below), corresponding to chart ID 1–84. To analyze the results, all glycans were ranked according to their signal-to-noise (S/N) ratio by dividing the mean RFU from four replicates by the mean background generated in the control wells lacking sialylated glycans. Variation within the 4 replicates was assessed as the coefficient of variation (%CV), which was calculated as 100 × S.D/Mean. Any value with a %CV of >30, was considered unacceptable as reported in Song et al. [40].

Abbreviations: LNnT = Galβ1-4GlcNAcβ1-3Galβ1-4Glc; LNT [Galβ1-3GlcNAcβ1-3Galβ1-4Glc]; NA2 = Galβ1-4GlcNAcβ1-2Manα1-6(Galβ1-4GlcNAcβ1-2Manα1-3)Manβ1-4GlcNAcβ1-4GlcNAc; Man5 = Manα1-6(Manα1-3)Manα1-6(Manα1-3)Manβ1-4GlcNAcβ1-4GlcNAc.

## Results

### Virus Purification

**Figure 1:**
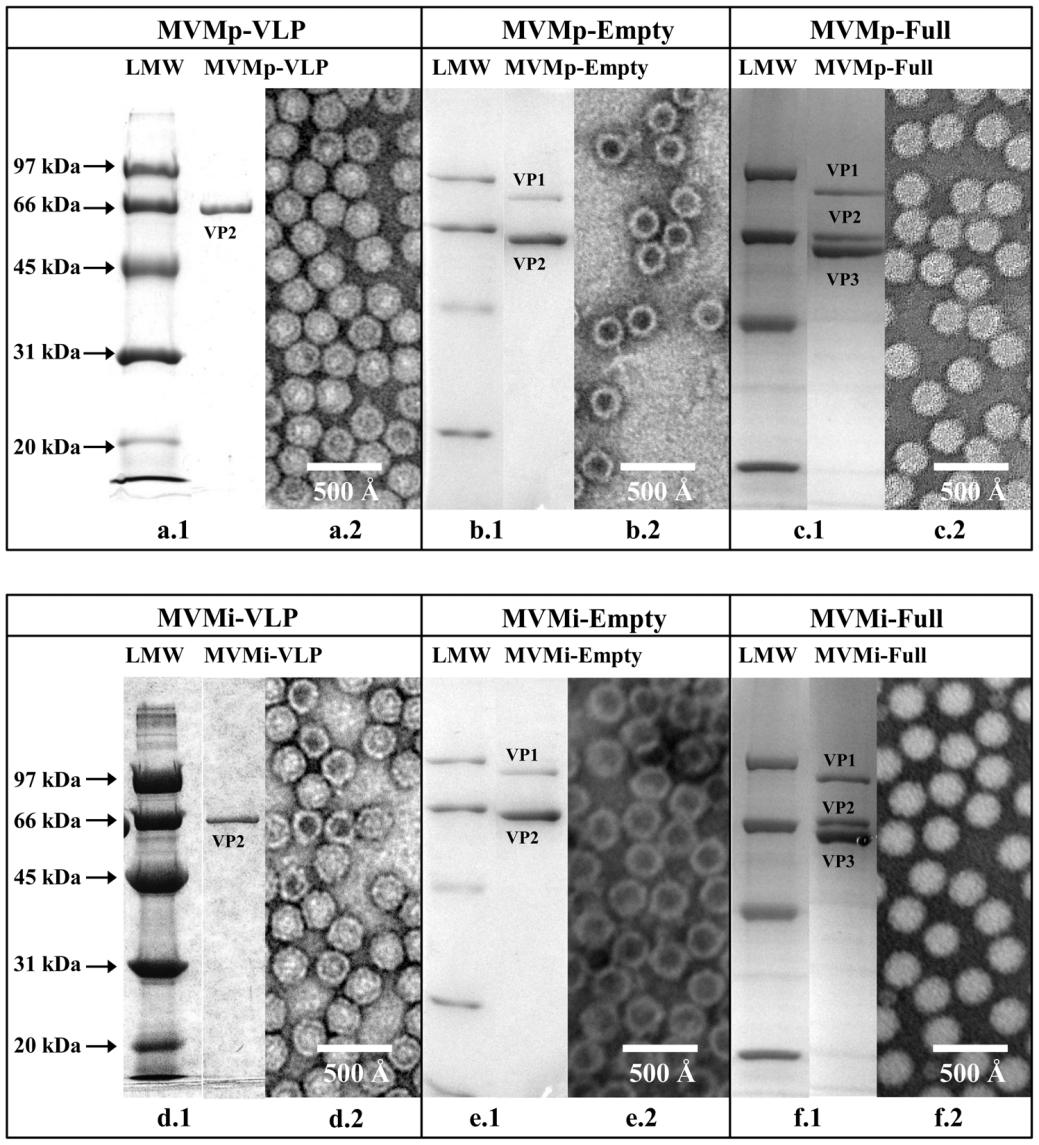
Virus purification. Coomassie stained SDS-PAGE for MVMp-VLP (a.1), MVMp-Empty (b.1), MVMp-Full (c.1), MVMi-VLP (d.1), MVMi-Empty (e.1), MVMi-Full (f.1). The positions of low-molecular-weight (LMW) standards (in kDa; Bio-Rad, Hercules, CA, USA) are indicated on the left-hand side. Negatively stained electron micrographs are shown in (a.2), (b.2), (c.2), (d.2), (e.2), and (f.2) respectively. Images in (b.2) and (c.2) were collected at 60,000X magnification while images in (a.2), (d.2), (e.2), and (f.2) were collected at 100,000X magnification.

The purity and integrity of the MVM capsids used in this study (MVMp-Full, MVMp-Empty, MVMp-VLP, MVMi-Full, MVMi-Empty, and MVMi-VLP) were verified by SDS-PAGE and negative-stain EM (Figure 1), prior to SGM screening. The SDS-PAGE analysis showed that the full capsids contained VP1 (83 kDa), VP2 (64 kDa), and VP3 (61 kDa); empty capsids contained VP1 and VP2; and all the VLPs contained only VP2. The negative stain EM analysis showed the full and empty capsids and VLPs to be intact.

### Recognition of Sialyl Derivatives by MVM Viruses on the SGM

**Figure 2:**
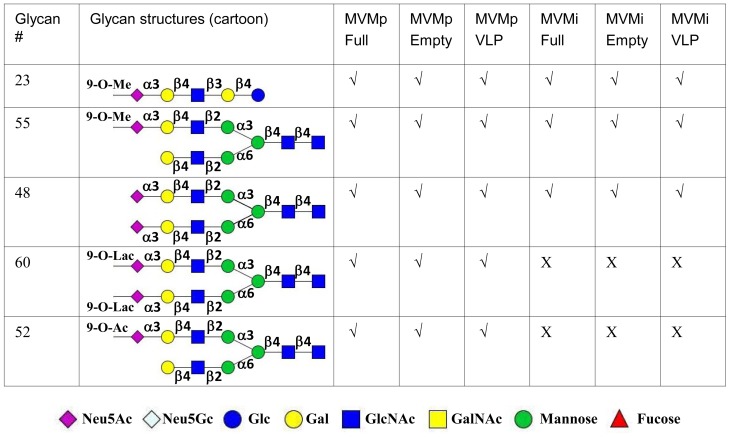
Sialylated derivatives recognized by the MVM viruses. The glycan number and the schematic representation are shown.

**Figure 3:**
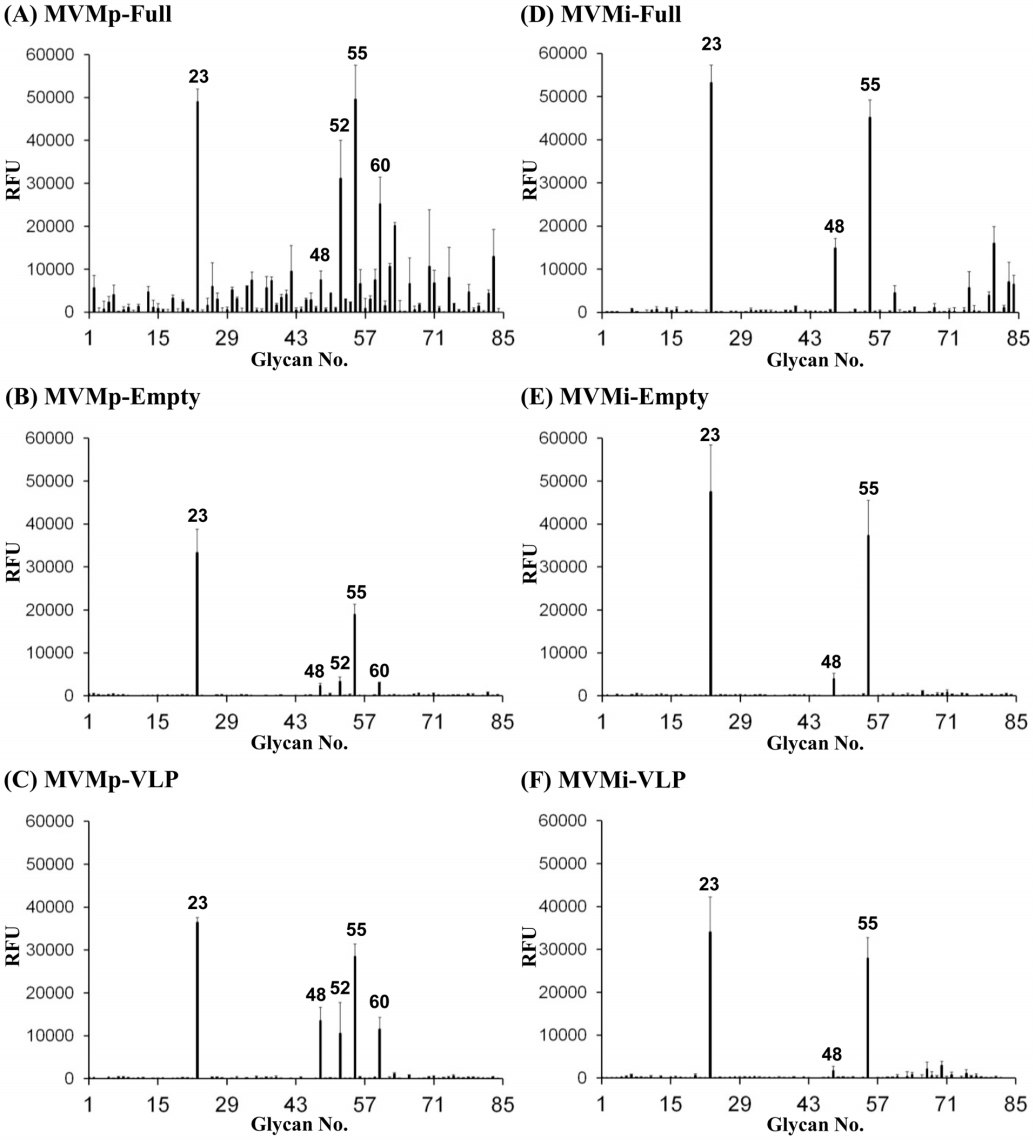
SGM array data for the MVM capsids. (A) MVMp-Full, (B) MVMp-Empty, (C) MVMp-VLP, (D) MVMi-Full, (E) MVMi-Empty, and (F) MVMi-VLP. The bars represent relative fluorescence for a given glycan. The glycans showing specificity to the MVM viruses are labeled.

Three types of MVMp and MVMi capsids; VLPs, empty, and full capsids, were screened on the SGM. For each particular strain, MVMp or MVMi, the binding profile was similar for the VLPs, empty, and full capsids (Figure 2). The SGM had both α2-3 and α2-6 linked sialylated glycans and 7 asialoglycans as controls, but MVMp and MVMi specifically bound only to the α2-3 linked sialylated derivatives (Figure 2 and 3; Supplemental Tables S2, S3, and S4). MVMp and MVMi showed a preference for binding to SIA that was methylated at C-9 and not C-8. Out of a total of 4 glycans that were derivatized with Neu5Ac9-O-methyl sialic acid, MVMp and MVMi bound only to two glycans, SGM 23 (Neu5Ac9Meα2-3Galβ1-4GlcNAcβ1-3Galβ1-4Glcitol) and SGM 55 (Neu5Ac9Meα2-3Galβ1-4GlcNAcβ1-2Manα1-3(Galβ1-4GlcNAcβ1-2 Manα1-6)Manβ1-4GlcNAcβ1-4GlcNAcitol), where the methylated sialic acid was α2-3 linked. The other two glycans with SIA that were methylated at C-9, SGM 8 (Neu5Ac9Meα2-6Galβ1-4GlcNAcβ1-3Galβ1-4Glcitol) and SGM 40 (Neu5Ac9Meα2-6Galβ1-4GlcNAcβ1-2Manα1-3(Galβ1-4GlcNAcβ1-2Manα1-6)Manβ1-4GlcNAcβ1-4GlcNAcitol) are similar to SGM 23 and SGM 55, respectively, but the sialic acid is α2-6 linked and not recognized by the MVM viruses. In addition, all the MVM viruses bound to the biantennary glycan, SGM 48 (Neu5Acα2-3Galβ1-4GlcNAcβ1-2Manα1-3(Neu5Acα2-3Galβ1-4GlcNAcβ1-2Manα1-6)Manβ1-4GlcNAcβ1-4GlcNAcitol) on the SGM.

All the MVMp viruses showed weak binding to two additional glycans that were not recognized by the MVMi viruses. The first was a biantennary glycan having Neu5,9Ac_2_ (9-O-acetylated SIA) where the sialic acid was linked α2-3 to the terminal Gal of type-2 glycan (SGM 52: Neu5,9Ac2α2-3Galβ1-4GlcNAcβ1-2Manα1-3(Galβ1-4GlcNAcβ1-2Manα1-6)Manβ1-4GlcNAcβ1-4GlcNAcitol). The second glycan had Neu5Ac9Lt (9-O-lactoylated SIA) where the sialic acid was linked α2-3 to the terminal Gal of type-2 glycan (SGM 60: Neu5Ac9Ltα2-3Galβ1-4GlcNAcβ1-2Manα1-3(Neu5Ac9Ltα2-3Galβ1-4GlcNAcβ1-2Manα1-6)Manβ1-4GlcNAcβ1-4GlcNAcitol). The binding profile for the genome containing viruses, especially MVMp virions, showed some weak non-specific binding, with high %CV, to other glycans compared to the VLPs and empty capsids (Figure 3). The affinity ranking of the derivatized glycans recognized by MVMp was in the order 9-O-methylated-monosialylated (SGM 23) >9-O-methylated-biantennary (SGM 55) >9-O-acetylated-biantennary (SGM 52) ≈ 9-O-lactolylated-biantennary (SGM 60) ≈ SIA-biantennary (SGM 48) (Figure 3). The affinity ranking for the MVMi viruses was 9-O-methylated-monosialylated (SGM 23) >9-O-methylated-biantennary (SGM 55)>SIA-biantennary (SGM 48). All these glycans had type-2 LacNAc (Galβ1-4GlcNAcβ1-R) as the core structure and were derived from either LNnT or NA2 precursor glycans. Consistent with the previous glycan array screening studies, none of the MVM viruses bound to the Neu5Gc or KDN sialylated derivatives [24], [35]. Notably, the SGM hits for three of the samples tested, MVMp-Empty, MVMp-VLP, and MVMi-VLP, were reproducible in a repeated screening (data not shown). The other samples were not re-screened due to limited availability.

### Glycan Profiling of Cell Lines Permissive/Restrictive for the MVM Viruses Validate Glycan Screening Data

**Figure 4:**
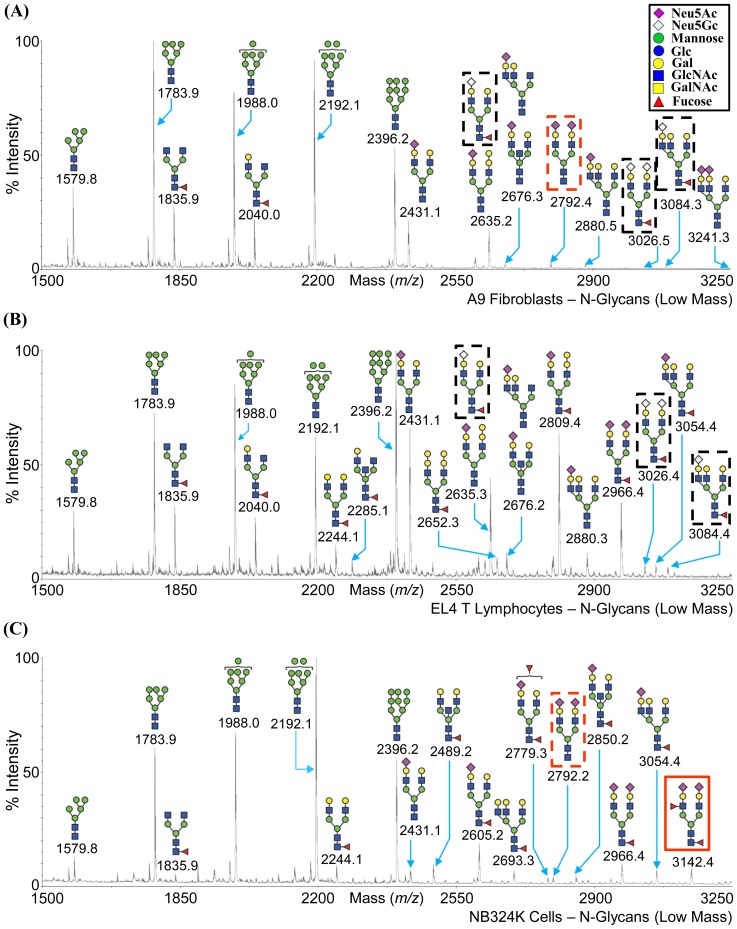
MALDI-TOF MS profiles of the permethylated low mass N-glycans. Low mass N-linked glycan expression on (A) A9 cells, (B) EL4 T cells, and (C) NB324K cells is shown. The glycans are annotated in the cartoon form. Inset depicts the cartoon legend.

**Figure 5:**
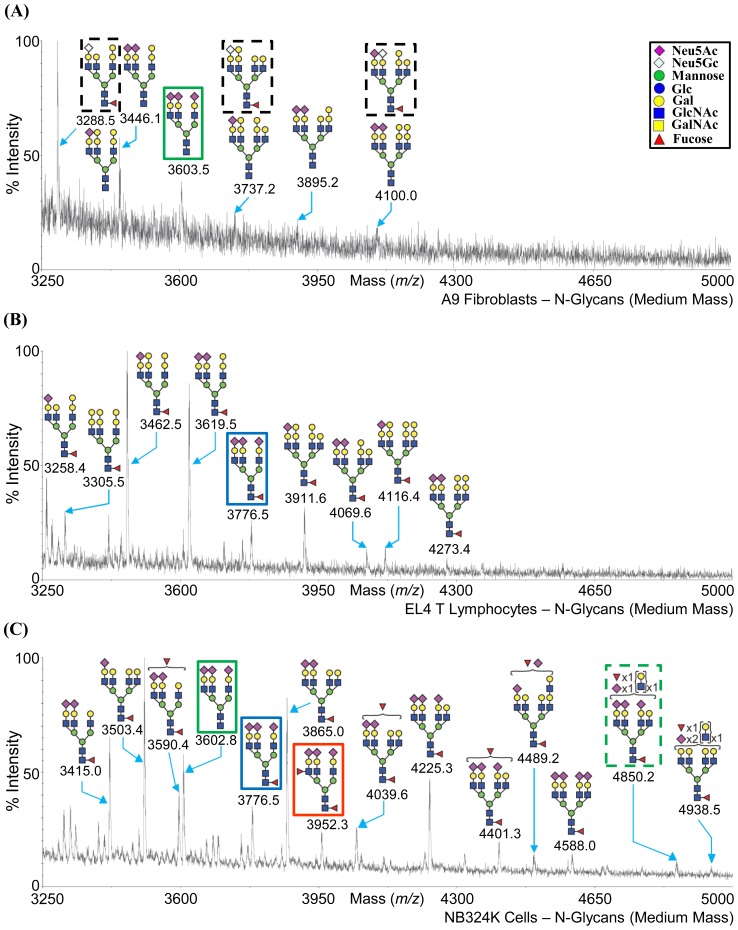
MALDI-TOF MS profiles of the permethylated medium mass N-glycans. Medium mass N-linked glycan expression on (A) A9 cells, (B) EL4 T cells, and (C) NB324K cells is shown. The glycans are annotated in the cartoon form. Inset depicts the cartoon legend.

**Figure 6:**
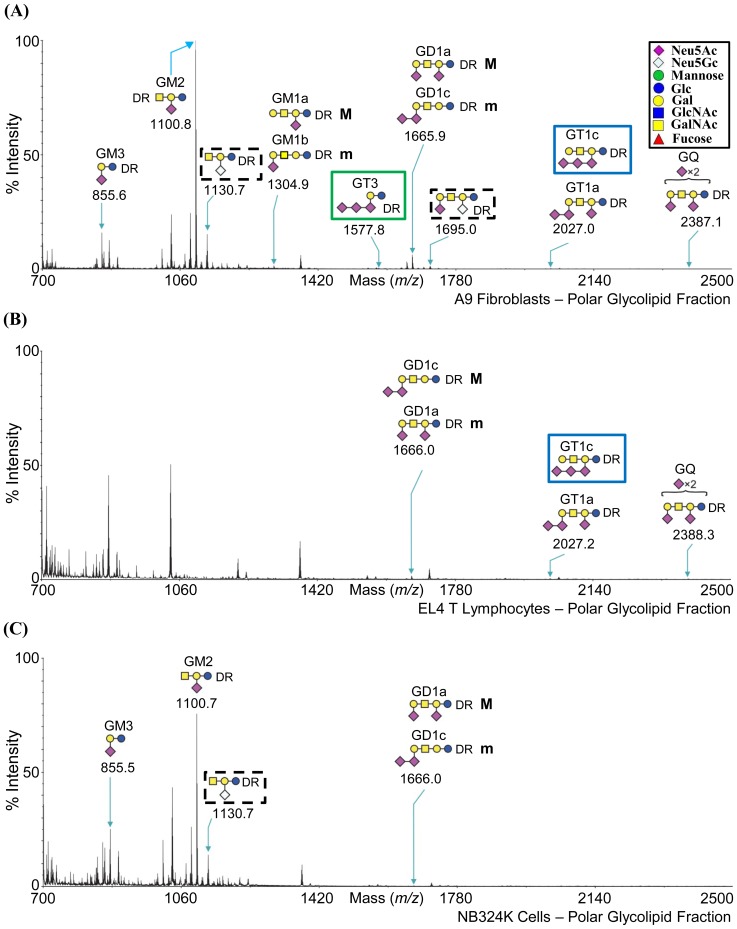
MALDI-TOF MS profiles of polar glycolipids. (A) A9 cells, (B) EL4 T cells, and (C) NB324K cells. The glycans are annotated in the cartoon form. The glycolipids were deutero-reduced (DR) to differentiate between symmetrical molecules in subsequent MS/MS analyses. M and m refer to major and minor species, respectively. Inset depicts the cartoon legend.

**Figure 7:**
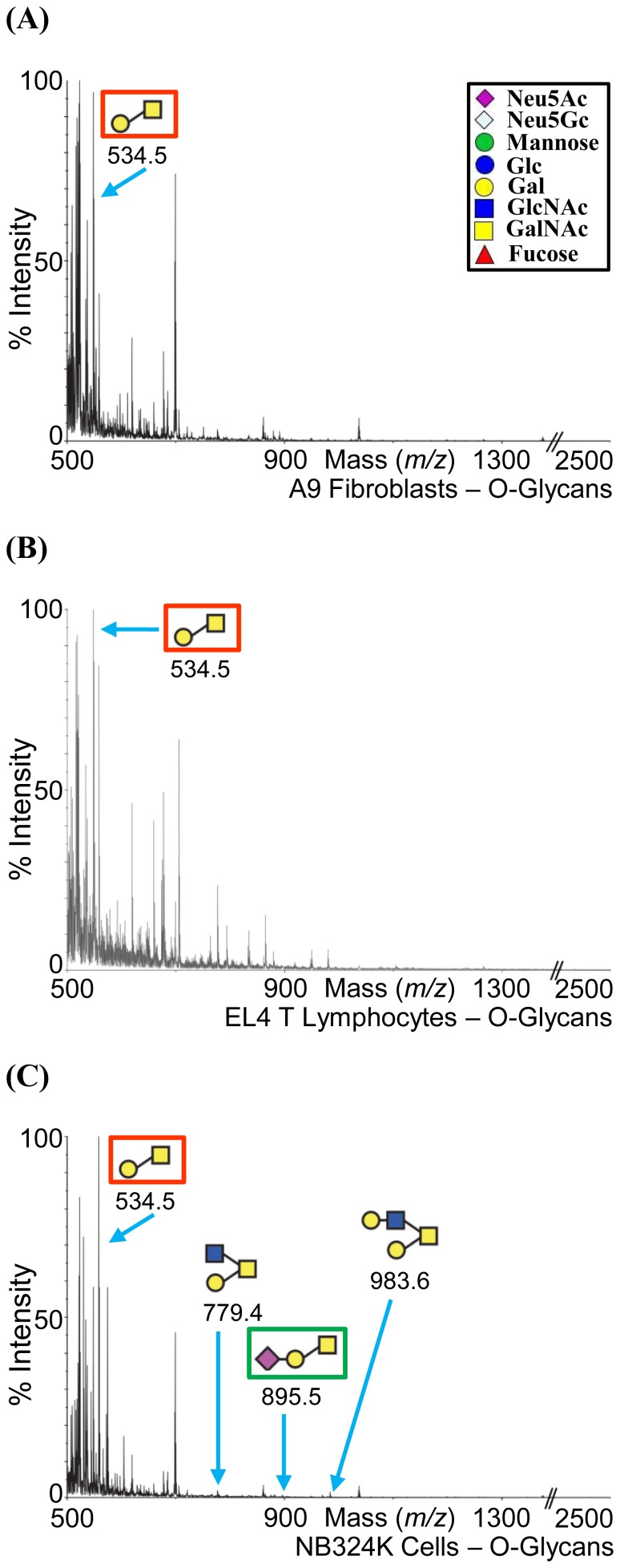
MALDI-TOF MS profiles of the O-glycans. (A) A9 cells, (B) EL4 T cells, and (C) NB324K cells. The glycans are annotated in the cartoon form. Inset depicts the cartoon legend.

Three cell lines, A9 fibroblasts (permissive for MVMp), EL4 T lymphocytes (permissive for MVMi), and NB324K transformed cells (permissive for both MVMp and MVMi) were subjected to glycomic profiling by MALDI-MS and MALDI-MS/MS analysis (http://www.functionalglycomics.org/glycomics/common/jsp/samples/searchSample.jsp?templateKey=2&12=CellType&operation=refine). The profiling provided information on the carbohydrate composition of the glycans present on these cells (Figures 4, 5, 6, and 7), but full linkages (α2-3SIA or α2-6SIA) present in the glycans was not determined. However, SIA-SIA (multisialylated) glycans can only be linked α2-8 or α2-9 to each other. Of the two derivatives of SIA, Neu5Ac is the major SIA in these cell lines, but Neu5Gc is present in trace amounts in the N-glycan fraction for A9 and EL4 T cells, and in the glycolipid fraction for the A9 and NB324K cells (enclosed in dashed black box in Figure 4A and B; Figure 5A; and Figure 6A and C). The single sialylated O-glycan observed in the NB324K cells had Neu5Ac (enclosed in green box in Figure 7C).

The mass spectra obtained from the peptide *N*-glycosidase F-released and permethylated *N*-glycans from these cell lines are shown in Figure 4 and 5. The N-glycans from all three cell lines showed a full complement of oligomannose as well as complex type N-glycans with bi-, tri-, or tetra-antennary structures. Hybrid N-glycans were not observed. The N-glycans on these cell lines terminated in Man, Gal, GlcNAc, or SIA. Structures consistent with presence of bisecting GlcNAc in complex N-glycans was also observed for all three cell lines. All the three cell lines also possessed core-fucosylated sialoglycans with the A9 cells showing the least expression of these glycans. In the NB324K cells only, a structure with a fucose linked to GlcNAc in the antennae, most likely as part of the 3′SIA-Le^x^ motif (Neu5Acα2-3Galβ1-4(Fucα1-3)GlcNAcβ) is observed (e.g., m/z = 3142.4, m/z = 3952.3, etc.; enclosed in red box in Figure 4C and 5C, respectively). The N-glycan composition for the A9 and EL4 T cells were very similar for the low molecular mass (Figure 4A and B), but for the medium mass there was more variation (Figure 5A and B). The NB324K cell line expresses more multi-antennary, poly lacNAc extended glycans than A9 and EL4 T cells (Figure 5C).

The profiles for the polar glycolipids are shown in Figure 6. For ease of identification, the common names (if known) of the identified glycolipids are included in Figure 6. The glycolipids present in the spectra are consistent with; GM3 (m/z = 855.5), GM2 (m/z = 1100.7), GM1a (m/z = 1304.9, major species), GM1b (m/z = 1304.9, minor species), GT3 (m/z = 1577.8), GD1a (m/z = 1665.9, major species), GD1c (m/z = 1665.9, minor species), GT1c/GT1a (m/z = 2027.0), and GQc/b (m/z = 2387.1) [41]. All the glycolipids were identified to be gangliosides (GalNAcβ1-4Galβ1-4GlcβCeramide motif). The multisialylated glycans (linked α2-8 or α2-9) are present in the polar glycolipid fraction for all the three cell lines. The ganglioside GT3, recognized by MVMi in a previous glycan array screening, was present only on A9 cells, at low abundance (enclosed in green box in Figure 6A) [24]. However, the core motif in GT3 (Neu5Acα-Neu5Acα-Neu5Acα-Gal-Glc) is also present in the GT1c that is expressed in EL4 T cells albeit also at low abundance (enclosed in blue box in Figure 6A and B). Branching of glycans was observed for the polar glycolipid fraction.

The mass spectra of the permethylated O-glycans from the three cell lines are shown in Figure 7. The ability to produce high quality O-glycan data after initial removal of N-glycans can be limited by low cell numbers. However, the A9 and EL4 T cells were shown to express a core 1 O-glycan, whilst the NB324K cells express both core 1 and core 2 O-glycans. The glycan that is expressed in all the three cell lines at the highest abundance (m/z = 534.5; enclosed in red box in Figure 7A–C) can be classified as T antigen and belongs to core 1 O-glycan (Galβ1-3GalNAcαSer/Thr) [42]. The only SIA containing O-glycan (green box in Figure 7C) is expressed on the NB324K cells and belongs to core 1. The remaining O-glycans expressed on the NB324K cells belong to core 2 (GlcNAcβ1-6(Galβ1-3)GalNAcαSer/Thr) (m/z = 779.4, m/z = 983.6).

## Discussion

### MVM Binds to Diverse Sialylated Derivatives

Glycan arrays attempt to mimic the diversity of glycans present on cell surfaces, and if a virus specifically recognizes a particular type of glycan on these arrays, it suggests that the virus may also recognize similar glycans in its natural host. The SGM binding profile for the three types of MVMp and MVMi capsids tested, VLPs, empty, and full capsids, were strain dependent (Figure 2 and 3). The recognition of similar glycans by full and empty capsids of each particular strain indicates that the encapsidation of DNA by the full capsids followed by its maturation, i.e. cleavage of VP2 to give VP3 [43], does not affect its receptor binding ability compared to empty capsids, consistent with previous observations. In addition, the similar recognition profile for empty capsids and VLPs supports the previous observations that they are antigenically and structurally equivalent [38], [44], [45].

The MVMp or MVMi viruses exhibited a specific preference for Neu5Acα2-3 glycans and did not bind to Neu5Gc or its derivatives even though it exists in mammals, and other parvoviruses such as AAV5 and CPV have been reported to recognize SIA glycans with this modification ([46], [47], and unpublished data). Also, none of the KDN derivatives were recognized, further confirming that the N-acetyl group at C-5 is preferred. The N-acetyl group at C-5 is within interaction distance of the side-chain of D399 and backbone of E400 in the MVM VP2 sequence. D399 has been shown to be an important determinant of SIA recognition by MVM and tissue tropism [23], [48].

The MVMp and MVMi viruses showed stronger binding to α2-3 SIA that is methylated at position C-9 of Neu5Ac (SGM 23 and SGM 55), compared to the other derivatives (acetylated or lactoylated Neu5Ac, such as SGM 52 and SGM 60, respectively, or Neu5Gc) and also non-derivatized Neu5Ac glycan (SGM 48) in the SGM (Figure 3). In addition, this hydrophobic methyl group is specifically preferred at position C-9 and not at C-8. This novel recognition is interesting, but its potential role in MVM infection is not known because this is a synthetic derivative that has not yet been observed in nature. Also, docking of a 3D model of a SIA glycan methylated at C-9 or C-8 of the glycerol side group into the MVMp SIA binding site localized to the icosahedral 2-fold region [23] did not provide any clues to this differential specificity (data not shown). The C-8 or C-9 of Neu5Ac in the MVMp-VLP-SIA complex structure is not in close proximity to any capsid surface amino acid residues that might affect the glycan binding interaction because the glycerol group points outward from the binding pocket (data not shown). MVMp and MVMi bound to the only Neu5Acα2-3 non-derivatized biantennary glycan on the SGM (SGM 48, Table S1). Thus while the signal was weak compared to the Neu5Ac methylated derivatives, recognition of SGM 48 suggests a strong preference for its structure which is consistent with the previous glycan array screenings [24], [35]. Significantly, this is consistent with the structures (based only on composition) observed in the cells that are permissive to MVM infections as determined by glycan profiling (Figure 4).

The MVMp viruses, but not MVMi viruses, bound to the 9-O-acetylated Neu5Ac derivative (SGM 52) (Figure 2, and Figure 3A, B and C). Significantly, SIA 9-O-acetylation is up-regulated (e.g., 9-O-acetylated GD3) in human melanoma cells [49]. The enveloped viruses bovine coronavirus (BCoV) and influenza c virus require 9-O-acetylated SIA containing receptor for a successful infection. These viruses possess an 9-O-acetyl esterase activity that promotes escape of virion progeny [50]. Thus it is possible that MVMp can also recognize and utilize this SIA derivative for the infection of cancer cells. The MVMp viruses also bound to the 9-O-lactoylated Neu5Ac derivative (SGM 60), which are present in serum glycoproteins [51]. A role for this interaction in MVMp infection is not obvious at this time. These additional MVMp interactions suggest that the surface properties of the MVMp capsid allows for the binding of SIA derivatives that are not recognized by the MVMi capsid and that the type of substitutions at position C-9 of the SIA dictated this extended recognition. However, despite the clustering of MVMp/MVMi amino acid differences at the 2-fold axis of the capsid [44] and based on the MVM-SIA complex structures currently available ([23], and unpublished data), the C-9 position of SIA derivatives is not involved in any capsid interactions, as discussed above. Thus a potential role for these recognition differences in dictating the MVMp and MVMi tissue tropism is not immediately evident and requires further study.

Previous glycan array screening studies had focused on identifying the oligosaccharide motif(s) recognized by MVM among a large number of different glycans [24] while screening on the SGM enabled the exploration of differential SIA-MVM strain recognition. MVM had been shown to bind to glycans with terminal Neu5Acα2-3 and not Neu5Acα2-6 [24]. The SGM screen confirmed these previous observations and the diversity of SIA modifications on this array allowed for detection of novel MVM glycan interactions, both naturally occurring and synthetic. In addition, this screen identified, for the first time, the recognition of biantennary glycans with terminal 3′SIA-LN motifs.

### Cellular Glycan Profiling Parallels Glycan Array Screening Data

The SIA (α2-3 or α2-6) and the LacNAc linkage information were not determined in the glycan profiling experiment. The glycan motifs recognized by MVM viruses in previous glycan arrays [24], [35] and current SGM (Figure 2), for example, SIA-LN (green or blue box in Figure 5A, B, and C), SIA-Le^x^ (red box in Figure 4C and 5C), and SIA-SIA-SIA (GT3, green box in Figure 6A), are also expressed on the three cell types profiled. In addition, the biantennary complex N-glycan, SGM 48, which was recognized by both viruses in the SGM array, is present in the low mass fraction of A9 cells and NB324K cells (m/z = 2792.2, enclosed in dashed red box in Figure 4A and C). The glycan SIA-LN-LN motif that was recognized in previous glycan array screening is possibly (based on the linkage combinations allowed) present in the NB324K medium mass N-glycan fraction (m/z = 4850.2) (enclosed in a dashed green box in Figure 5C). As with the SGM, the three cell lines displayed a variety of carbohydrates, including Neu5Ac, Neu5Gc, and non-sialylated glycans. This observation thus validates the use of such arrays to identify potential binding partners for macromolecules, including viruses.

The polar glycolipid fractions of A9 and EL4 T cells, but not their N- and O-glycan fractions, showed the presence of α2-8 multisialylated glycans, such as GT3 which were recognized by MVMi viruses, but not MVMp, in a previous glycan array screening [24]. The restricted infection of A9 cells by MVMi is thus likely not controlled by this glycan interaction. Previous experiments have shown that a SIA containing glycoprotein (and not glycolipid) is utilized as a receptor for MVM infection [7], [23], but the glycan array utilized by Nam et al. [24] did not differentiate the origin of the GT3. Several recent studies have shown that α2-8 multisialylated glycans, especially GT3 and GD3, are present on glycoproteins in the mouse brain [34], [52]–[54]. Thus it is possible that MVMi viruses utilize an α2-8 multisialylated glycoprotein for infection in neuronal cells. Multisialylated glycoproteins with α2-8 linkages, such as Neural Cell Adhesion Molecule (NCAM), MCAM (melanoma cell adhesion molecule), and CD166 (or Activated Leukocyte Cell Adhesion Molecule (ALCAM)), are commonly found expressed on the neuronal cells and could serve as receptors for MVMi on these cells [53], [55]. A profiling of a mouse derived neuronal cell line would aid in validating the abundance of and the possibility that MVMi utilizes α2-8 linked glycans for neuronal cell infection.

The NB324K cell line is a transformed cell line that expresses sialylated N-glycans with the SIA-Le^x^ motif (e.g., m/z = 3142.4, m/z = 3952.3, etc.; enclosed in red box in Figure 4C and 5C, respectively) which is a known carbohydrate marker for cancer cells [32], [33], [56], [57]. The expression of this motif has also been specifically observed in the N-glycan profile of other transformed cell lines such as THP-1 (monocytic leukemia cell line), HL-60 (promyelocytic leukemia cells), and K562 (erythromyeloblastoid leukemia cell line) (http://www.functionalglycomics.org/glycomics/publicdata/glycoprofiling-new.jsp). This motif was recognized previously by the MVM viruses on a glycan array [24]. This supports the suggestion that the SIA-Le^x^ motif on tumor cell lines might be utilized by the oncotropic MVM viruses for infection.

The A9 and EL4 T cells have a similar glycan profile and an abundance of sialylated glycans recognized by the two viruses in glycan arrays. This similarity in glycan profile thus fails to explain the differences in tropism for these cells between these MVM viruses based on differential cell surface glycan receptor recognition. These observations support previous claims that the determinant of differential cell and tissue tropism for MVMp and MVMi is post cell entry [26], [28], [36], [58], [59]. These previous reports identified amino acids at and surrounding the depression at icosahedral 2-fold axes of the capsid, which differ between the viruses, as playing a crucial role in dictating these differences. The site reported for the MVMp-SIA interaction is a pocket flanking the 2-fold axis [23]. The observed similarity in glycan recognition by the two viruses suggests that the critical glycan interacting residue(s) is conserved. It is possible to envisage a scenario in which, following the initial capsid interaction with a cell surface glycan, an MVM strain specific interaction with a cellular host factor present only in the permissive cell line, initiates downstream events required for infection. These post entry interactions could also be under the control of similar glycan(s) on host cell specific glycoprotein receptors yet to be identified. These possibilities remain to be verified.

### Summary

For the MVM viruses, both modifications of SIA and their linkage to core structures regulate recognition. The binding profile for VLPs, empty, and full particles was similar on the SGM which validates the use of VLPs in lieu of infectious virions for structural and biochemical studies that examine receptor interactions. Future directions include structural studies of the MVM virus complexed with the glycans identified in microarrays to elucidate the capsid-receptor interactions that likely dictate the similarities and differences observed for MVMp and MVMi. The glycomic profiling data correlates with the glycans that were recognized by the MVM viruses in a previous glycan screening and those identified in the current SGM, such as glycans with SIA-LN motif, SIA-Le^x^ motif, the α2,8 multisialylated glycans, and the biantennary glycans. The abundance and variability of SIA containing glycans on these cell surfaces, makes them suitable receptors for numerous interactions, as exemplified by the large number of viruses, such as MVM, for which this interaction is essential for infection.

## Supporting Information

Table S1 **The chart ID and glycan structures of the sialylated glycan microarray (SGM).** Structures with an asterisk are monosialylated biantennary glycans.
(DOC)
