# Supplementary material for: Profiling of Glycan Receptors for Minute Virus of Mice in Permissive Cell Lines Towards Understanding the Mechanism of Cell Recognition
Source: PLoS One. 2014 Jan 27;9(1):e86909. doi: 10.1371/journal.pone.0086909 (PMC3903596; doi:10.1371/journal.pone.0086909)
Supplement: Table S1 — The chart ID and glycan structures of the sialylated glycan microarray (SGM). Structures with an asterisk are monosialylated biantennary glycans. (DOC) [file pone.0086909.s001.doc]

Supplemental Table S1. The chart ID and glycan structures of the sialylated glycan microarray (SGM). Structures with an asterisk are monosialylated biantennary glycans.

| Glycan No. | Structure |
| --- | --- |
| 1 | Neu5Acα6Galβ4GlcNAcβ3Galβ4Glcitol-AEAB |
| 2 | Neu5Ac8Me α6Galβ4GlcNAcβ3Galβ4Glcitol-AEAB |
| 3 | Neu5Gc α6Galβ4GlcNAcβ3Galβ4Glcitol-AEAB |
| 4 | Kdnα6Galβ4GlcNAcβ3Galβ4Glcitol-AEAB |
| 5 | Neu5,9Ac2α6Galβ4GlcNAcβ3Galβ4Glcitol-AEAB |
| 6 | Neu5,9Gc2α6Galβ4GlcNAcβ3Galβ4Glcitol-AEAB |
| 7 | Kdn9Acα6Galβ4GlcNAcβ3Galβ4Glcitol-AEAB |
| 8 | Neu5Ac9Meα6Galβ4GlcNAcβ3Galβ4Glcitol-AEAB |
| 9 | Neu5GcOMeα6Galβ4GlcNAcβ3Galβ4Glcitol-AEAB |
| 10 | Kdn9Meα6Galβ4GlcNAcβ3Galβ4Glcitol-AEAB |
| 11 | Kdn7Meα6Galβ4GlcNAcβ3Galβ4Glcitol-AEAB |
| 12 | Neu5Ac9Ltα6Galβ4GlcNAcβ3Galβ4Glcitol-AEAB |
| 13 | Neu5GcOAcα6Galβ4GlcNAcβ3Galβ4Glcitol-AEAB |
| 14 | Kdn5Acα6Galβ4GlcNAcβ3Galβ4Glcitol-AEAB |
| 15 | Kdn5,9Ac2α6Galβ4GlcNAcβ3Galβ4Glcitol-AEAB |
| 16 | Neu5Acα3Galβ4GlcNAcβ3Galβ4Glcitol-AEAB |
| 17 | Neu5Ac8Meα3Galβ4GlcNAcβ3Galβ4Glcitol-AEAB |
| 18 | Neu5Gcα3Galβ4GlcNAcβ3Galβ4Glcitol-AEAB |
| 19 | Kdnα3Galβ4GlcNAcβ3Galβ4Glcitol-AEAB |
| 20 | Neu5,9Ac2α3Galβ4GlcNAcβ3Galβ4Glcitol-AEAB |
| 21 | Neu5,9Gc2α3Galβ4GlcNAcβ3Galβ4Glcitol-AEAB |
| 22 | Kdn9Acα3Galβ4GlcNAcβ3Galβ4Glcitol-AEAB |
| 23 | Neu5Ac9Meα3Galβ4GlcNAcβ3Galβ4Glcitol-AEAB |
| 24 | Neu5GcOMeα3Galβ4GlcNAcβ3Galβ4Glcitol-AEAB |
| 25 | Kdn5Meα3Galβ4GlcNAcβ3Galβ4Glcitol-AEAB |
| 26 | Kdn7Meα3Galβ4GlcNAcβ3Galβ4Glcitol-AEAB |
| 27 | Neu5Ac9Ltα3Galβ4GlcNAcβ3Galβ4Glcitol-AEAB |
| 28 | Neu5GcOAcα3Galβ4GlcNAcβ3Galβ4Glcitol-AEAB |
| 29 | Kdn5Acα3Galβ4GlcNAcβ3Galβ4Glcitol-AEAB |
| 30 | Kdn5,9Ac2α3Galβ4GlcNAcβ3Galβ4Glcitol-AEAB |
| 31 | Neu5Acα6Galβ4Glcitol-AEAB |
| 32 | Neu5Gcα6Galβ4Glcitol-AEAB |
| 33 | Neu5Acα3Galβ4Glcitol-AEAB |
| 34 | Neu5Gcα3Galβ4Glcitol-AEAB |
| 35 | Neu5Acα6Galβ4GlcNAcβ2Manα3(Neu5Acα6Galβ4GlcNAcβ2Manα6)Manβ4GlcNAcβ4GlcNAcitol-AEAB |
| 36 | Neu5Ac8Meα6Galβ4GlcNAcβ2Manα3(Neu5Ac8Meα6Galβ4GlcNAcβ2Manα6)Manβ4GlcNAcβ4GlcNAcitol-AEAB |
| 37 | Neu5Gcα6Galβ4GlcNAcβ2Manα3(Neu5Gcα6Galβ4GlcNAcβ2Manα6)Manβ4GlcNAcβ4GlcNAcitol-AEAB |
| 38 | Neu5,9Ac2α6Galβ4GlcNAcβ2Manα3(Neu5,9Ac2α6Galβ4GlcNAcβ2Manα6)Manβ4GlcNAcβ4GlcNAcitol-AEAB |
| 39 | Neu5Gc9Acα6Galβ4GlcNAcβ2Manα3(Neu5Gc9Acα6Galβ4GlcNAcβ2Manα6)Manβ4GlcNAcβ4GlcNAcitol-AEAB |
| 40 | *Neu5Ac9Meα6Galβ4GlcNAcβ2Manα3(Galβ4GlcNAcβ2Manα6)Manβ4GlcNAcβ4GlcNAcitol-AEAB |
| 41 | Neu5GcOMeα6Galβ4GlcNAcβ2Manα3(Neu5GcOMeα6Galβ4GlcNAcβ2Manα6)Manβ4GlcNAcβ4GlcNAcitol-AEAB |
| 42 | *Kdn9Meα6Galβ4GlcNAcβ2Manα3(Galβ4GlcNAcβ2Manα6)Manβ4GlcNAcβ4GlcNAcitol-AEAB |
| 43 | *Kdn5Meα6Galβ4GlcNAcβ2Manα3(Galβ4GlcNAcβ2Manα6)Manβ4GlcNAcβ4GlcNAcitol-AEAB |
| 44 | *Kdn7Meα6Galβ4GlcNAcβ2Manα3(Galβ4GlcNAcβ2Manα6)Manβ4GlcNAcβ4GlcNAcitol-AEAB |
| 45 | Neu5Ac9Ltα6Galβ4GlcNAcβ2Manα3(Neu5Ac9Ltα6Galβ4GlcNAcβ2Manα6)Manβ4GlcNAcβ4GlcNAcitol-AEAB |
| 46 | Neu5GcOAcα6Galβ4GlcNAcβ2Manα3(Neu5GcOAcα6Galβ4GlcNAcβ2Manα6)Manβ4GlcNAcβ4GlcNAcitol-AEAB |
| 47 | Kdn5Acα6Galβ4GlcNAcβ2Manα3(Kdn5Acα6Galβ4GlcNAcβ2Manα6)Manβ4GlcNAcβ4GlcNAcitol-AEAB |
| 48 | Neu5Acα3Galβ4GlcNAcβ2Manα3(Neu5Acα3Galβ4GlcNAcβ2Manα6)Manβ4GlcNAcβ4GlcNAcitol-AEAB |
| 49 | *Neu5Ac8Meα3Galβ4GlcNAcβ2Manα3(Galβ4GlcNAcβ2Manα6)Manβ4GlcNAcβ4GlcNAcitol-AEAB |
| 50 | Neu5Gcα3Galβ4GlcNAcβ2Manα3(Neu5Gcα3Galβ4GlcNAcβ2Manα6)Manβ4GlcNAcβ4GlcNAcitol-AEAB |
| 51 | Kdnα3Galβ4GlcNAcβ2Manα3(Kdnα3Galβ4GlcNAcβ2Manα6)Manβ4GlcNAcβ4GlcNAcitol-AEAB |
| 52 | * Neu5,9Ac2α3Galβ4GlcNAcβ2Manα3(Galβ4GlcNAcβ2Manα6)Manβ4GlcNAcβ4GlcNAcitol-AEAB |
| 53 | Neu5Gc9Acα3Galβ4GlcNAcβ2Manα3(Neu5Gc9Acα3Galβ4GlcNAcβ2Manα6)Manβ4GlcNAcβ4GlcNAcitol-AEAB |
| 54 | * Kdn9Acα3Galβ4GlcNAcβ2Manα3(Galβ4GlcNAcβ2Manα6)Manβ4GlcNAcβ4GlcNAcitol-AEAB |
| 55 | *Neu5Ac9Meα3Galβ4GlcNAcβ2Manα3(Galβ4GlcNAcβ2Manα6)Manβ4GlcNAcβ4GlcNAcitol-AEAB |
| 56 | Neu5GcOMeα3Galβ4GlcNAcβ2Manα3Neu5GcOMeα3Galβ4GlcNAcβ2Manα6)Manβ4GlcNAcβ4GlcNAcitol-AEAB |
| 57 | *Kdn9Meα3Galβ4GlcNAcβ2Manα3(Galβ4GlcNAcβ2Manα6)Manβ4GlcNAcβ4GlcNAcitol-AEAB |
| 58 | *Kdn5Meα3Galβ4GlcNAcβ2Manα3(Galβ4GlcNAcβ2Manα6)Manβ4GlcNAcβ4GlcNAcitol-AEAB |
| 59 | *Kdn7Meα3Galβ4GlcNAcβ2Manα3(Galβ4GlcNAcβ2Manα6)Manβ4GlcNAcβ4GlcNAcitol-AEAB |
| 60 | Neu5Ac9Ltα3Galβ4GlcNAcβ2Manα3(Neu5Ac9Ltα3Galβ4GlcNAcβ2Manα6)Manβ4GlcNAcβ4GlcNAcitol-AEAB |
| 61 | *Neu5GcOAcα3Galβ4GlcNAcβ2Manα3(Galβ4GlcNAcβ2Manα6)Manβ4GlcNAcβ4GlcNAcitol-AEAB |
| 62 | Kdn5Acα3Galβ4GlcNAcβ2Manα3(Kdn5Acα3Galβ4GlcNAcβ2Manα6)Manβ4GlcNAcβ4GlcNAcitol-AEAB |
| 63 | Neu5Acα3Galβ3GlcNAcβ3Galβ4Glcitol-AEAB |
| 64 | Neu5Gcα3Galβ3GlcNAcβ3Galβ4Glcitol-AEAB |
| 65 | Neu5,9Ac2α3Galβ3GlcNAcβ3Galβ4Glcitol-AEAB |
| 66 | Kdn9Acα3Galβ3GlcNAcβ3Galβ4Glcitol-AEAB |
| 67 | Neu5GcOMeα3Galβ3GlcNAcβ3Galβ4Glcitol-AEAB |
| 68 | Neu5Acα6Galβ3GlcNAcβ3Galβ4Glcitol-AEAB |
| 69 | Neu5Gc α6Galβ3GlcNAcβ3Galβ4Glcitol-AEAB |
| 70 | Kdnα6Galβ3GlcNAcβ3Galβ4Glcitol-AEAB |
| 71 | Neu5,9Ac2α6Galβ3GlcNAcβ3Galβ4Glcitol-AEAB |
| 72 | Kdn9Acα6Galβ3GlcNAcβ3Galβ4Glcitol-AEAB |
| 73 | Neu5GcOMeα6Galβ3GlcNAcβ3Galβ4Glcitol-AEAB |
| 74 | Kdn5Meα6Galβ3GlcNAcβ3Galβ4Glcitol-AEAB |
| 75 | Neu5Ac9Ltα6Galβ3GlcNAcβ3Galβ4Glcitol-AEAB |
| 76 | Neu5GcOAcα6Galβ3GlcNAcβ3Galβ4Glcitol-AEAB |
| 77 | Kdn5Acα6Galβ3GlcNAcβ3Galβ4Glcitol-AEAB |
| 78 | Galβ4GlcNAcβ3Galβ4Glcitol-AEAB |
| 79 | Galβ4GlcNAcβ2Manα3(Galβ4GlcNAcβ2Manα6)Manβ4GlcNAcβ4GlcNAcitol-AEAB |
| 80 | Manα1-6(Manα1-3)Manα1-6(Manα1-3)Manβ1-4GlcNAcβ1-4 GlcNAcitol-AEAB |
| 81 | Galβ3GlcNAcβ3Galβ4Glcitol-AEAB |
| 82 | Galβ4Glcitol-AEAB |
| 83 | Fetuin-AEAB |
| 84 | Biotin-AEAB |

Supplemental Table S2. The binding of MVMi viruses to the sialylated glycan microarray

| Glycan No. | MVMi-Full | | | MVMi-Empty | | | MVMi-VLP | | |
| --- | --- | --- | --- | --- | --- | --- | --- | --- | --- |
|  | Average RFU | STDEV | %CV | Average RFU | STDEV | %CV | Average RFU | STDEV | %CV |
| 1 | -684 | 187 | -27 | -86 | 303 | -355 | 75 | 116 | 155 |
| 2 | -152 | 383 | -252 | 194 | 15 | 8 | 88 | 10 | 11 |
| 3 | 34 | 171 | 507 | -160 | 153 | -96 | 3 | 82 | 2718 |
| 4 | -101 | 333 | -329 | 324 | 42 | 13 | 12 | 132 | 1103 |
| 5 | -202 | 192 | -95 | 175 | 26 | 15 | 245 | 23 | 10 |
| 6 | -191 | 119 | -62 | -88 | 76 | -86 | 312 | 213 | 68 |
| 7 | 712 | 226 | 32 | 260 | 52 | 20 | 788 | 93 | 12 |
| 8 | 4 | 214 | 4945 | 355 | 257 | 73 | 111 | 137 | 124 |
| 9 | -645 | 522 | -81 | 128 | 312 | 244 | 88 | 273 | 309 |
| 10 | -126 | 630 | -502 | 2 | 55 | 2433 | -13 | 172 | -1324 |
| 11 | 515 | 29 | 6 | 110 | 61 | 56 | 238 | 338 | 142 |
| 12 | 673 | 664 | 99 | 211 | 138 | 66 | -2 | 92 | -6121 |
| 13 | -55 | 220 | -404 | 222 | 262 | 118 | 247 | 258 | 105 |
| 14 | 744 | 318 | 43 | 202 | 70 | 35 | -47 | 147 | -315 |
| 15 | 321 | 95 | 30 | 94 | 51 | 54 | 236 | 44 | 19 |
| 16 | 769 | 514 | 67 | 217 | 57 | 26 | 151 | 273 | 180 |
| 17 | -48 | 69 | -143 | 239 | 57 | 24 | 165 | 2 | 1 |
| 18 | 327 | 60 | 18 | -84 | 90 | -108 | 46 | 77 | 169 |
| 19 | 189 | 363 | 193 | -27 | 319 | -1170 | 69 | 38 | 55 |
| 20 | -131 | 296 | -225 | 93 | 81 | 87 | 601 | 403 | 67 |
| 21 | -163 | 137 | -84 | 70 | 51 | 73 | 30 | 70 | 237 |
| 22 | 86 | 415 | 481 | 192 | 10 | 5 | 66 | 42 | 64 |
| 23 | 53224 | 4063 | 8 | 47524 | 10868 | 23 | 34001 | 8176 | 24 |
| 24 | -44 | 256 | -585 | 59 | 74 | 125 | 50 | 61 | 121 |
| 25 | 133 | 87 | 65 | 262 | 92 | 35 | 188 | 122 | 65 |
| 26 | -345 | 171 | -50 | 128 | 119 | 93 | 47 | 81 | 171 |
| 27 | 216 | 81 | 38 | 83 | 121 | 146 | 188 | 26 | 14 |
| 28 | 205 | 42 | 21 | 245 | 51 | 21 | 252 | 53 | 21 |
| 29 | -123 | 278 | -226 | 146 | 187 | 128 | -553 | 865 | -157 |
| 30 | 128 | 98 | 77 | 184 | 6 | 3 | 125 | 188 | 150 |
| 31 | 567 | 403 | 71 | 90 | 31 | 35 | 232 | 35 | 15 |
| 32 | 364 | 8 | 2 | 93 | 180 | 195 | 319 | 49 | 15 |
| 33 | 305 | 237 | 78 | 309 | 52 | 17 | -14 | 341 | -2481 |
| 34 | 329 | 197 | 60 | 164 | 104 | 63 | 106 | 92 | 87 |
| 35 | 109 | 396 | 363 | -57 | 18 | -32 | 158 | 47 | 30 |
| 36 | 189 | 49 | 26 | 99 | 27 | 27 | 67 | 40 | 60 |
| 37 | -99 | 244 | -247 | -137 | 253 | -185 | -383 | 699 | -183 |
| 38 | 462 | 96 | 21 | 29 | 29 | 98 | 142 | 37 | 26 |
| 39 | 344 | 98 | 29 | 181 | 33 | 18 | 84 | 7 | 8 |
| 40 | 1412 | 100 | 7 | 194 | 105 | 54 | 288 | 45 | 15 |
| 41 | 50 | 27 | 54 | 148 | 23 | 15 | -984 | 564 | -57 |

| Glycan No. | MVMi-Full | | | MVMi-Empty | | | MVMi-VLP | | |
| --- | --- | --- | --- | --- | --- | --- | --- | --- | --- |
|  | Average RFU | STDEV | %CV | Average RFU | STDEV | %CV | Average RFU | STDEV | %CV |
| 42 | 207 | 358 | 173 | 240 | 70 | 29 | 236 | 43 | 18 |
| 43 | 199 | 85 | 43 | 88 | 45 | 51 | 57 | 9 | 16 |
| 44 | 289 | 23 | 8 | -7 | 213 | -3153 | -79 | 81 | -103 |
| 45 | 135 | 41 | 30 | 0 | 100 | 29895 | -96 | 136 | -142 |
| 46 | -109 | 301 | -277 | -72 | 132 | -182 | 140 | 27 | 19 |
| 47 | 617 | 74 | 12 | -44 | 159 | -360 | 187 | 35 | 19 |
| 48 | 14842 | 2288 | 15 | 3949 | 1281 | 32 | 1693 | 1090 | 64 |
| 49 | -36 | 79 | -222 | -50 | 27 | -53 | 183 | 88 | 48 |
| 50 | -35 | 91 | -262 | 115 | 59 | 51 | 258 | 82 | 32 |
| 51 | 82 | 78 | 96 | -7 | 206 | -2942 | 149 | 27 | 18 |
| 52 | 602 | 155 | 26 | 84 | 104 | 124 | 184 | 177 | 96 |
| 53 | -39 | 197 | -506 | 56 | 94 | 169 | -48 | 194 | -408 |
| 54 | 195 | 66 | 34 | 312 | 176 | 56 | 187 | 1 | 1 |
| 55 | 45118 | 4110 | 9 | 37344 | 8190 | 22 | 27902 | 4828 | 17 |
| 56 | 122 | 158 | 130 | -92 | 205 | -223 | -172 | 229 | -133 |
| 57 | 178 | 319 | 180 | 76 | 51 | 68 | -202 | 394 | -195 |
| 58 | -100 | 170 | -170 | -72 | 460 | -644 | 94 | 48 | 51 |
| 59 | 211 | 164 | 77 | 21 | 122 | 579 | -28 | 313 | -1129 |
| 60 | 4525 | 1676 | 37 | 299 | 310 | 104 | -379 | 634 | -167 |
| 61 | -273 | 921 | -338 | 28 | 2 | 8 | 329 | 537 | 163 |
| 62 | -76 | 255 | -334 | 10 | 288 | 2885 | -160 | 300 | -187 |
| 63 | 198 | 187 | 95 | 170 | 458 | 270 | 371 | 1128 | 304 |
| 64 | 1116 | 137 | 12 | 105 | 192 | 183 | 804 | 507 | 63 |
| 65 | -47 | 115 | -245 | 11 | 52 | 473 | -4 | 17 | -406 |
| 66 | -8 | 4 | -47 | 1022 | 193 | 19 | -773 | 1508 | -195 |
| 67 | 121 | 72 | 60 | -109 | 361 | -331 | 2112 | 1638 | 78 |
| 68 | 1120 | 942 | 84 | 88 | 167 | 190 | 622 | 877 | 141 |
| 69 | 107 | 12 | 11 | 335 | 381 | 114 | 194 | 377 | 194 |
| 70 | -121 | 313 | -260 | 596 | 69 | 12 | 2912 | 952 | 33 |
| 71 | 392 | 420 | 107 | 819 | 551 | 67 | -830 | 1300 | -157 |
| 72 | 181 | 894 | 495 | 375 | 18 | 5 | 772 | 623 | 81 |
| 73 | -52 | 105 | -201 | 108 | 76 | 71 | -4 | 59 | -1601 |
| 74 | 437 | 616 | 141 | 474 | 197 | 42 | 78 | 316 | 408 |
| 75 | 5682 | 3766 | 66 | 342 | 158 | 46 | 1095 | 878 | 80 |
| 76 | 398 | 1140 | 286 | -133 | 71 | -53 | 314 | 263 | 84 |
| 77 | 153 | 145 | 95 | 76 | 17 | 23 | 521 | 447 | 86 |
| 78 | -791 | 909 | -115 | 263 | 180 | 69 | 283 | 107 | 38 |
| 79 | 3936 | 851 | 22 | 74 | 51 | 69 | 83 | 98 | 118 |
| 80 | 15994 | 3893 | 24 | 328 | 159 | 49 | 149 | 35 | 23 |
| 81 | 80 | 89 | 111 | 48 | 17 | 35 | 169 | 293 | 173 |
| 82 | 1051 | 602 | 57 | 266 | 46 | 17 | 19 | 87 | 470 |
| 83 | 7047 | 4571 | 65 | 362 | 223 | 62 | -40 | 88 | -221 |
| 84 | 6529 | 2027 | 31 | 156 | 162 | 104 | -25 | 40 | -160 |

Supplemental Table S3. The binding of MVMp viruses to the sialylated glycan microarray

| Glycan No. | MVMp-Full | | | MVMp-Empty | | | MVMp-VLP | | |
| --- | --- | --- | --- | --- | --- | --- | --- | --- | --- |
|  | Average RFU | STDEV | %CV | Average RFU | STDEV | %CV | Average RFU | STDEV | %CV |
| 1 | -2327 | 1367 | -59 | 401 | 107 | 27 | 338 | 32 | 9 |
| 2 | 5640 | 2880 | 51 | 491 | 108 | 22 | 261 | 36 | 14 |
| 3 | -377 | 1431 | -380 | 164 | 194 | 118 | -77 | 225 | -294 |
| 4 | 667 | 1856 | 278 | -759 | 985 | -130 | -19 | 50 | -270 |
| 5 | 2298 | 1323 | 58 | 286 | 30 | 10 | 352 | 23 | 7 |
| 6 | 4058 | 2228 | 55 | 420 | 95 | 23 | 101 | 87 | 87 |
| 7 | -33 | 265 | -816 | 171 | 54 | 32 | 249 | 265 | 106 |
| 8 | 520 | 732 | 141 | 14 | 300 | 2143 | 310 | 196 | 63 |
| 9 | 1078 | 745 | 69 | 91 | 59 | 64 | 226 | 144 | 64 |
| 10 | -21 | 499 | -2432 | -31 | 54 | -177 | 50 | 96 | 193 |
| 11 | 1361 | 467 | 34 | -11 | 91 | -827 | 172 | 45 | 26 |
| 12 | -1147 | 861 | -75 | 32 | 11 | 33 | 237 | 122 | 52 |
| 13 | 4708 | 1295 | 28 | 55 | 22 | 40 | -57 | 108 | -189 |
| 14 | 1112 | 1656 | 149 | 99 | 37 | 38 | 107 | 82 | 77 |
| 15 | 876 | 1068 | 122 | 158 | 70 | 44 | 38 | 156 | 409 |
| 16 | 438 | 240 | 55 | 40 | 4 | 11 | 158 | 28 | 18 |
| 17 | -718 | 1366 | -190 | 170 | 42 | 25 | -65 | 95 | -147 |
| 18 | 3298 | 692 | 21 | 67 | 50 | 74 | 27 | 16 | 61 |
| 19 | -767 | 1511 | -197 | -12 | 174 | -1488 | 98 | 54 | 55 |
| 20 | 2367 | 524 | 22 | 304 | 42 | 14 | 219 | 139 | 64 |
| 21 | 679 | 167 | 25 | 119 | 119 | 100 | 173 | 93 | 53 |
| 22 | 311 | 158 | 51 | 2 | 47 | 3158 | 49 | 50 | 100 |
| 23 | 49037 | 2962 | 6 | 33287 | 5470 | 16 | 36478 | 1080 | 3 |
| 24 | 84 | 486 | 582 | 66 | 40 | 60 | -8 | 84 | -1095 |
| 25 | 1550 | 1723 | 111 | -30 | 20 | -67 | -78 | 50 | -64 |
| 26 | 6016 | 5525 | 92 | -2 | 22 | -1296 | 342 | 124 | 36 |
| 27 | 3040 | 1438 | 47 | 210 | 30 | 14 | 287 | 158 | 55 |
| 28 | -156 | 1176 | -753 | 258 | 59 | 23 | -65 | 353 | -545 |
| 29 | 590 | 616 | 105 | 79 | 49 | 62 | -33 | 42 | -125 |
| 30 | 5191 | 602 | 12 | 83 | 46 | 56 | 136 | 24 | 18 |
| 31 | 3128 | 376 | 12 | 8 | 111 | 1442 | 230 | 315 | 137 |
| 32 | -30 | 987 | -3289 | 304 | 57 | 19 | -98 | 52 | -53 |
| 33 | 5988 | 120 | 2 | 122 | 168 | 137 | 143 | 158 | 110 |
| 34 | 7428 | 1938 | 26 | 96 | 16 | 16 | NA | NA | NA |
| 35 | 447 | 480 | 107 | -68 | 83 | -122 | 348 | 211 | 61 |
| 36 | 181 | 555 | 307 | -42 | 117 | -278 | 13 | 153 | 1177 |
| 37 | 5677 | 2599 | 46 | -25 | 222 | -898 | 325 | 76 | 23 |
| 38 | 7323 | 908 | 12 | -33 | 96 | -292 | -31 | 375 | -1201 |
| 39 | 1646 | 448 | 27 | 41 | 77 | 185 | 269 | 385 | 143 |
| 40 | 3413 | 737 | 22 | 170 | 120 | 70 | 70 | 87 | 124 |
| 41 | 4144 | 942 | 23 | -25 | 21 | -84 | -78 | 121 | -155 |

| Glycan No. | MVMp-Full | | | MVMp-Empty | | | MVMp-VLP | | |
| --- | --- | --- | --- | --- | --- | --- | --- | --- | --- |
|  | Average RFU | STDEV | %CV | Average RFU | STDEV | %CV | Average RFU | STDEV | %CV |
| 42 | 9482 | 6038 | 64 | -48 | 176 | -369 | 147 | 16 | 11 |
| 43 | 416 | 632 | 152 | 49 | 40 | 81 | -42 | 166 | -398 |
| 44 | 700 | 474 | 68 | -36 | 182 | -508 | 287 | 162 | 57 |
| 45 | 2846 | 400 | 14 | 181 | 47 | 26 | -167 | 247 | -148 |
| 46 | 2862 | 1589 | 56 | 90 | 148 | 165 | -11 | 112 | -1022 |
| 47 | 1035 | 299 | 29 | -97 | 73 | -76 | 58 | 63 | 109 |
| 48 | 7490 | 2131 | 28 | 2390 | 531 | 22 | 13488 | 3140 | 23 |
| 49 | 594 | 424 | 71 | 77 | 84 | 110 | -265 | 474 | -179 |
| 50 | 4423 | 50 | 1 | 386 | 187 | 48 | 217 | 21 | 10 |
| 51 | 878 | 204 | 23 | -230 | 223 | -97 | 120 | 58 | 49 |
| 52 | 31143 | 8926 | 29 | 3341 | 1044 | 31 | 10559 | 7168 | 68 |
| 53 | 2968 | 155 | 5 | 42 | 166 | 398 | 38 | 81 | 217 |
| 54 | 2321 | 93 | 4 | 223 | 243 | 109 | 89 | 62 | 70 |
| 55 | 49574 | 7965 | 16 | 18927 | 2402 | 13 | 28503 | 2921 | 10 |
| 56 | 6665 | 3277 | 49 | 103 | 19 | 18 | 355 | 117 | 33 |
| 57 | 822 | 2374 | 289 | 61 | 18 | 30 | 28 | 49 | 178 |
| 58 | 2995 | 862 | 29 | -62 | 62 | -99 | -116 | 334 | -289 |
| 59 | 7508 | 2487 | 33 | 64 | 228 | 354 | 320 | 52 | 16 |
| 60 | 25181 | 6315 | 25 | 3109 | 63 | 2 | 11518 | 2758 | 24 |
| 61 | 1511 | 1101 | 73 | 38 | 10 | 27 | -88 | 176 | -200 |
| 62 | 10607 | 754 | 7 | 190 | 10 | 5 | 131 | 330 | 252 |
| 63 | 20155 | 713 | 4 | 140 | 184 | 131 | 1040 | 336 | 32 |
| 64 | 242 | 2483 | 1025 | 144 | 81 | 56 | -128 | 340 | -266 |
| 65 | 61 | 124 | 204 | 17 | 2 | 13 | -34 | 101 | -293 |
| 66 | 6656 | 6013 | 90 | 143 | 67 | 46 | 817 | 95 | 12 |
| 67 | 558 | 869 | 156 | 389 | 78 | 20 | -119 | 203 | -170 |
| 68 | 1776 | 276 | 16 | 526 | 132 | 25 | 10 | 3 | 31 |
| 69 | 156 | 142 | 91 | 97 | 29 | 30 | -13 | 172 | -1353 |
| 70 | 10598 | 13257 | 125 | 169 | 23 | 13 | 413 | 94 | 23 |
| 71 | 6790 | 2954 | 44 | 451 | 210 | 46 | 431 | 275 | 64 |
| 72 | 910 | 452 | 50 | 181 | 7 | 4 | 48 | 31 | 64 |
| 73 | 17 | 1 | 8 | 7 | 2 | 21 | 142 | 194 | 136 |
| 74 | 8042 | 7080 | 88 | 51 | 105 | 205 | 288 | 200 | 70 |
| 75 | 1979 | 81 | 4 | 215 | 28 | 13 | 627 | 275 | 44 |
| 76 | 477 | 156 | 33 | -22 | 309 | -1387 | -84 | 425 | -505 |
| 77 | 100 | 105 | 105 | 11 | 3 | 26 | 177 | 129 | 73 |
| 78 | 4738 | 1752 | 37 | 413 | 82 | 20 | 279 | 154 | 55 |
| 79 | 563 | 487 | 86 | 222 | 279 | 126 | 237 | 217 | 92 |
| 80 | 1384 | 695 | 50 | -32 | 31 | -96 | -3 | 321 | -9634 |
| 81 | 84 | 259 | 309 | 19 | 10 | 51 | 186 | 32 | 17 |
| 82 | 4354 | 862 | 20 | 698 | 175 | 25 | 134 | 41 | 31 |
| 83 | 12962 | 6308 | 49 | -41 | 200 | -484 | 453 | 79 | 18 |
| 84 | -645 | 1506 | -233 | 182 | 129 | 71 | 61 | 86 | 142 |

Supplemental Table S4. The binding of MVM viruses to the SGM (Biological replicate data)

| Glycan No. | MVMp-Empty | | | MVMp-VLP | | | MVMi-VLP | | |
| --- | --- | --- | --- | --- | --- | --- | --- | --- | --- |
|  | Average RFU | STDEV | %CV | Average RFU | STDEV | %CV | Average RFU | STDEV | %CV |
| 1 | 24 | 11 | 45 | 116 | 19 | 17 | 118 | 5 | 4 |
| 2 | 9 | 7 | 83 | 109 | 20 | 18 | 69 | 44 | 64 |
| 3 | 15 | 3 | 21 | 52 | 35 | 68 | 12 | 56 | 486 |
| 4 | 10 | 3 | 28 | 33 | 15 | 47 | 46 | 7 | 15 |
| 5 | 15 | 1 | 5 | -47 | 44 | -92 | -32 | 139 | -435 |
| 6 | 4 | 2 | 61 | -7 | 33 | -502 | -243 | 135 | -56 |
| 7 | 4 | 4 | 93 | 27 | 10 | 36 | 57 | 3 | 6 |
| 8 | 17 | 5 | 28 | 42 | 7 | 17 | 17 | 32 | 193 |
| 9 | -13 | 21 | -167 | -5 | 43 | -865 | 206 | 161 | 78 |
| 10 | 144 | 3 | 2 | -11 | 28 | -246 | -25 | 75 | -299 |
| 11 | 54 | 10 | 19 | -2 | 9 | -503 | 30 | 7 | 24 |
| 12 | 86 | 32 | 37 | 259 | 199 | 77 | -46 | 70 | -152 |
| 13 | 83 | 12 | 15 | 5 | 12 | 229 | 29 | 52 | 176 |
| 14 | 92 | 29 | 31 | 18 | 11 | 61 | -17 | 55 | -320 |
| 15 | 114 | 17 | 15 | 51 | 17 | 33 | -39 | 93 | -241 |
| 16 | 365 | 96 | 26 | 178 | 12 | 6 | -74 | 43 | -58 |
| 17 | 53 | 15 | 28 | 21 | 52 | 248 | -196 | 395 | -202 |
| 18 | 179 | 22 | 12 | 39 | 10 | 26 | -6 | 70 | -1217 |
| 19 | 143 | 31 | 22 | 86 | 20 | 24 | -62 | 52 | -83 |
| 20 | 585 | 82 | 14 | 26 | 2 | 7 | 215 | 41 | 19 |
| 21 | 132 | 73 | 55 | 31 | 2 | 6 | 115 | 141 | 123 |
| 22 | 39 | 18 | 45 | 38 | 15 | 39 | -47 | 69 | -147 |
| 23 | 11990 | 1434 | 12 | 12728 | 755 | 6 | 15728 | 1063 | 7 |
| 24 | 92 | 87 | 95 | 22 | 8 | 38 | 33 | 37 | 111 |
| 25 | 117 | 14 | 12 | 16 | 31 | 191 | 102 | 28 | 27 |
| 26 | 190 | 59 | 31 | 67 | 9 | 13 | -7 | 30 | -452 |
| 27 | 288 | 29 | 10 | 81 | 46 | 56 | 97 | 46 | 47 |
| 28 | 109 | 32 | 29 | 2 | 15 | 903 | -14 | 68 | -495 |
| 29 | 70 | 19 | 27 | 8 | 22 | 292 | -12 | 149 | -1220 |
| 30 | 130 | 38 | 29 | 16 | 12 | 78 | -1 | 10 | -1480 |
| 31 | 404 | 66 | 16 | 133 | 28 | 21 | 457 | 75 | 16 |
| 32 | 117 | 33 | 28 | 69 | 20 | 30 | 109 | 119 | 109 |
| 33 | 699 | 128 | 18 | 231 | 58 | 25 | 413 | 63 | 15 |
| 34 | 178 | 49 | 28 | 63 | 20 | 32 | 28 | 34 | 121 |
| 35 | 215 | 30 | 14 | 107 | 18 | 17 | 648 | 96 | 15 |
| 36 | 35 | 14 | 39 | 27 | 10 | 36 | -3 | 174 | -6951 |
| 37 | 40 | 25 | 63 | 98 | 11 | 11 | 211 | 130 | 62 |
| 38 | 600 | 10 | 2 | 272 | 82 | 30 | 461 | 129 | 28 |
| 39 | 41 | 3 | 7 | 41 | 11 | 26 | 120 | 104 | 86 |
| 40 | 370 | 33 | 9 | 143 | 22 | 15 | 179 | 17 | 9 |
| 41 | 35 | 15 | 44 | 13 | 4 | 33 | 220 | 33 | 15 |

| Glycan No. | MVMp-Empty | | | MVMp-VLP | | | MVMi-VLP | | |
| --- | --- | --- | --- | --- | --- | --- | --- | --- | --- |
|  | Average RFU | STDEV | %CV | Average RFU | STDEV | %CV | Average RFU | STDEV | %CV |
| 42 | 134 | 44 | 33 | 58 | 43 | 74 | 351 | 97 | 28 |
| 43 | 79 | 21 | 27 | 76 | 40 | 53 | 290 | 12 | 4 |
| 44 | 41 | 7 | 16 | 67 | 23 | 33 | -68 | 211 | -310 |
| 45 | 204 | 23 | 11 | 50 | 14 | 28 | 34 | 30 | 89 |
| 46 | 112 | 11 | 10 | 42 | 18 | 44 | 136 | 22 | 16 |
| 47 | 61 | 23 | 39 | 24 | 4 | 14 | 28 | 55 | 197 |
| 48 | 6121 | 77 | 1 | 8965 | 1599 | 18 | 1474 | 356 | 24 |
| 49 | 179 | 48 | 27 | 37 | 17 | 47 | 256 | 71 | 28 |
| 50 | 93 | 5 | 5 | 35 | 9 | 26 | 65 | 14 | 22 |
| 51 | 26 | 9 | 35 | 0 | 13 | NA | 239 | 128 | 54 |
| 52 | 2145 | 191 | 9 | 5609 | 1328 | 24 | 62 | 17 | 28 |
| 53 | 74 | 19 | 26 | 68 | 26 | 38 | 77 | 78 | 103 |
| 54 | 47 | 15 | 32 | 13 | 2 | 16 | 59 | 14 | 24 |
| 55 | 12728 | 641 | 5 | 14395 | 1116 | 8 | 16823 | 2120 | 13 |
| 56 | 6 | 1 | 24 | 10 | 2 | 20 | 89 | 58 | 65 |
| 57 | 75 | 8 | 11 | 109 | 19 | 17 | 518 | 355 | 68 |
| 58 | 72 | 17 | 24 | 14 | 5 | 36 | 73 | 23 | 32 |
| 59 | 16 | 13 | 86 | 5 | 5 | 92 | 115 | 53 | 46 |
| 60 | 1974 | 473 | 24 | 793 | 181 | 23 | -33 | 96 | -287 |
| 61 | 24 | 11 | 45 | -14 | 38 | -279 | 68 | 33 | 48 |
| 62 | 40 | 13 | 34 | 18 | 9 | 49 | 8 | 12 | 140 |
| 63 | 138 | 35 | 26 | 134 | 28 | 21 | 326 | 108 | 33 |
| 64 | 11 | 6 | 57 | 9 | 5 | 59 | -81 | 70 | -87 |
| 65 | 24 | 14 | 58 | -8 | 10 | -124 | -11 | 266 | -2473 |
| 66 | 15 | 6 | 40 | 44 | 9 | 20 | -14 | 73 | -519 |
| 67 | 6 | 8 | 144 | 19 | 4 | 22 | 10 | 96 | 1011 |
| 68 | 32 | 16 | 49 | 25 | 15 | 59 | 10 | 18 | 183 |
| 69 | 13 | 3 | 23 | 42 | 10 | 23 | 142 | 114 | 80 |
| 70 | 13 | 3 | 22 | 41 | 13 | 31 | -80 | 88 | -110 |
| 71 | 95 | 30 | 32 | 101 | 63 | 63 | 217 | 68 | 31 |
| 72 | 18 | 4 | 21 | 10 | 0 | 0 | -43 | 42 | -99 |
| 73 | 32 | 11 | 33 | 13 | 8 | 62 | 524 | 299 | 57 |
| 74 | 27 | 6 | 22 | -9 | 29 | -311 | -18 | 214 | -1190 |
| 75 | 69 | 6 | 9 | -23 | 56 | -242 | -78 | 178 | -229 |
| 76 | 14 | 4 | 31 | 40 | 34 | 87 | -59 | 106 | -180 |
| 77 | 78 | 73 | 93 | 7 | 3 | 40 | 70 | 62 | 89 |
| 78 | -9 | 9 | -97 | 12 | 3 | 25 | 70 | 17 | 24 |
| 79 | 3 | 1 | 35 | 16 | 0 | 0 | -311 | 300 | -96 |
| 80 | 9 | 7 | 77 | 8 | 3 | 33 | -110 | 124 | -113 |
| 81 | 13 | 5 | 38 | 14 | 9 | 59 | 88 | 17 | 19 |
| 82 | 35 | 9 | 26 | 15 | 4 | 26 | 54 | 40 | 75 |
| 83 | 415 | 120 | 29 | 503 | 94 | 19 | 586 | 59 | 10 |
| 84 | 6 | 3 | 48 | 13 | 2 | 11 | 3 | 36 | 1184 |
